# Early transcriptional changes of heavy metal resistance and multiple efflux genes in *Xanthomonas campestris* pv. *campestris* under copper and heavy metal ion stress

**DOI:** 10.1186/s12866-024-03206-7

**Published:** 2024-03-09

**Authors:** Stephen D. B. Ramnarine, Omar Ali, Jayaraj Jayaraman, Adesh Ramsubhag

**Affiliations:** Department of Life Sciences, Faculty of Science and Technology, The University of The West Indies, St. Augustine campus, St. Augustine, Trinidad and Tobago W. I

**Keywords:** Oxidative stress, Copper tolerance, Copper stress, Heavy metal stress, RNA-seq, Transcriptome, *Xanthomonas campestris* pv. *campestris*, MDR, Efflux pumps, *Cop*

## Abstract

**Background:**

Copper-induced gene expression in *Xanthomonas campestris* pv. *campestris* (Xcc) is typically evaluated using targeted approaches involving qPCR. The global response to copper stress in Xcc and resistance to metal induced damage is not well understood. However, homologs of heavy metal efflux genes from the related *Stenotrophomonas* genus are found in *Xanthomonas* which suggests that metal related efflux may also be present.

**Methods and Results:**

Gene expression in Xcc strain BrA1 exposed to 0.8 mM CuSO_4_.5H_2_O for 15 minutes was captured using RNA-seq analysis. Changes in expression was noted for genes related to general stress responses and oxidoreductases, biofilm formation, protein folding chaperones, heat-shock proteins, membrane lipid profile, multiple drug and efflux (MDR) transporters, and DNA repair were documented. At this timepoint only the *cohL* (copper homeostasis/tolerance) gene was upregulated as well as a chromosomal *czcCBA* efflux operon. An additional screen up to 4 hrs using qPCR was conducted using a wider range of heavy metals. Target genes included a *cop-*containing heavy metal resistance island and putative metal efflux genes. Several efflux pumps, including a copper resistance associated homolog from *S. maltophilia*, were upregulated under toxic copper stress. However, these pumps were also upregulated in response to other toxic heavy metals. Additionally, the temporal expression of the *coh* and *cop* operons was also observed, demonstrating co-expression of tolerance responses and later activation of part of the *cop* operon.

**Conclusions:**

Overall, initial transcriptional responses focused on combating oxidative stress, mitigating protein damage and potentially increasing resistance to heavy metals and other biocides. A putative copper responsive efflux gene and others which might play a role in broader heavy metal resistance were also identified. Furthermore, the expression patterns of the *cop* operon in conjunction with other copper responsive genes allowed for a better understanding of the fate of copper ions in *Xanthomonas.* This work provides useful evidence for further evaluating MDR and other efflux pumps in metal-specific homeostasis and tolerance phenotypes in the *Xanthomonas* genus. Furthermore, non-canonical copper tolerance and resistance efflux pumps were potentially identified. These findings have implications for interpreting MIC differences among strains with homologous *copLAB* resistance genes, understanding survival under copper stress, and resistance in disease management.

**Supplementary Information:**

The online version contains supplementary material available at 10.1186/s12866-024-03206-7.

## Introduction

Copper resistance in *Xanthomonas campestris* pv. *campestris* (Xcc) is strongly attributed to plasmid-borne *copLAB* genes. A shift to a more copper tolerant/resistant *Xanthomonas* population associated with agricultural fields in Trinidad and, genome-phenotype characteristics of multi-resistance have been documented in local strains (Ramnarine et al, Unpublished). While chromosomal *cohLAB* genes are implicated in copper homeostasis/tolerance responses [1, 2], their function appears to be homologous to *copLAB* resistance genes and differ in regulation only [3]. Questions still remain on how *Xanthomonas* can deal with metal ion-induced stress and copper translocation out of the cytoplasm in the initial phases of exposure. Localization of excess metal ions in the periplasmic space via cytoplasmic efflux represents one major response to toxic metal stress [4], where mitigation strategies are typically concentrated [5]. These responses are facilitated by efflux pumps of the RND, CDF and P-Type ATPase families [6] usually targeting specific metal ions. However, except for one *Xanthomonas* strain [7], no efflux system specifically associated with basal copper tolerance responses has been characterised.

Often, as a part of the first line of active defence mechanisms, multidrug efflux (MDR) pumps confer broad resistance to antibiotics and other xenobiotics [8]. Some of these efflux pump categories include SMR (small multidrug resistance), ABC (ATP-Binding cassette), MFS (Multi-Facilitator Superfamily) and, the RND (Resistance-Nodulation-Cell division superfamily). These protein families are involved in import and export functions for a broad range of substrates including heavy metals [9, 10] such as the metal-specific *czcCBA* genes responsible for Co, Zn and Cd export of the RND superfamily. Extensive characterisation of homologous protein families in the closely related *Stenotrophomonas* genus provides multiple MDR and putative metal efflux gene targets in *Xanthomonas* spp. Furthermore, a link to enhanced MDR (multi-drug resistance) efflux pump activity in response to heavy metal exposure in *Pseudomonas* sp. highlights the coordinated and broad response of bacterial cells to single stress factors [11, 12]. Anthropogenic activities such as the use of heavy metal-containing agrochemicals and industrial pollution serve to enrich and further adapt these mechanisms towards specialized responses [13, 14]. However, there is a dearth of evidence on other potential efflux mechanisms in response to copper stress in *Xanthomonas* spp.

Our previous study on resistome characterisation of local *Xanthomonas* strains demonstrated varying MIC’s to high concentrations of heavy metals and antibiotics in conjunction with the presence of a heavy metal resistance island and efflux pump homologs (Ramnarine et al, Unpublished). These findings informed the current study which sought to reveal the early molecular responses to copper stress in a local Trinidadian Xcc strain (BrA1) isolated by Lugo et al. (2013) [15]. To this end RNA-seq was employed to identify putative targets for enrichment studies. Particular focus was made to the heavy metal resistance island, the *coh* operon and the gene neighbourhood surrounding these loci. Furthermore, the expression of efflux pumps at the initial stage was also documented. These targets were then chosen for further evaluation in another local Xcc strain (Cf4B1) using qPCR at later timepoints of exposure and using different heavy metals to measure their sustained co-expression under differnet stress conditions.

A hybrid transcript analysis pipeline involving both reference mapping and Trinity De-novo-based assembly revealed numerous responses priming cells for oxidative damage control. Furthermore, the expression profiles of qPCR targets provided insights into the co-expression and temporal response of the *copLABMGF* operon and, downstream heavy metal resistance and efflux pump genes. Some efflux pump targets were only expressed at higher heavy metal concentrations, suggesting potentially new targets for copper and other heavy metal resistance evaluation in *Xanthomonas*. The findings of this study reveal the broader initial molecular response mechanisms to copper resistance, heavy metal stress and the potential priming of bacterial cells for broader resistance capabilities through broad-spectrum MDR pump activity.

## Methods

### Xcc strain BrA1 culture conditions for RNA-seq

Xcc strain BrA1 previously isolated [15] was sub-cultured onto mannitol-glutamate-yeast (MGY) broth amended with 0.08 mM CuSO_4_.5H_2_O (induction medium) [1] to induce copper-associated gene expression over 24h. Induced cultures were multiplied in 100ml of induction medium over 24h to an OD_600_ of 0.6. Ten ml aliquots were pelleted and resuspended in MGY broth amended with a sub-lethal but still toxic level of CuSO_4_.5H_2_O (0.8 mM) to induce copper stress in bacterial cells. A 15-minute exposure period was chosen to capture early gene expression changes. After incubation at 35°C for this time point, cells were pelleted and immediately frozen in liquid nitrogen. Unamended MGY broth was used as a control. Total RNA was extracted using TRIzol (Invitrogen) according to the manufacturer’s protocols. The RNA samples were assessed using a NanoDrop 2000 and 1% Agarose gel [16] and stored at -80°C for eventual Illumina sequencing.

### Illumina sequencing of Xcc BrA1 RNA and data analysis

DNase treatment, RNA quality assessment, library construction and QC, and Illumina sequencing were carried out at Novogene Corporation INC (USA). RNA samples with RIN value ≥ 6 (Agilent 2100 Bioanalyzer) were carried forward for rRNA depletion (Ribo-Zero TM Magnetic kit) and Library preparation (NEBNext® Ultra™ II Directional RNA Kit), and quality assessment and normalization via qPCR. Illumina sequencing (HiSeq 2500) followed (150bp PE, 250-350bp insert size) and fastq were files obtained from the sequencing centre. Raw reads were uploaded to the NCBI SRA under Bioproject: PRJNA756283. FastQC, Read mapping, transcript assembly and counts, and annotation were done using a hybrid approach on the public domain Galaxy server [17], OMICSbox v1.4.12 [18] and command-line tools. Initial QC of reads was carried out using FastQC v0.72 [19] and trimmed using Trimmomatic v0.38 [20] in PE mode to retain read pair association and Phred score >30.

Sample and read quality metrics are given in Supplemental Table 1. Reference-based read mapping and counts were obtained using HISAT2 v2.1.0 [21], featureCounts v2.0.1 [22], the RefSeq genome and, manually formatted GTF file of Xcc ATCC 33913 (NC_003902.1). Read pair association of unmapped reads were maintained using Trimmomatic and assembled using the Trinity v2.10.0 [23] de-novo based approach with Super-Transcripts construction enabled. Predicted ORFs and CDS were determined using TransDecoder v5.5.0 [24] and counts (CPM) were obtained using the included RSEM [25] package, in OmicsBox. Differential gene expression between copper stress and control contrasts was calculated using edgeR v3.24.1 [26] with trimmed mean of means (TMM) normalization and counts per million (CPM) filter of 1 in at least 1 sample. Significant differentially expressed genes (DEG’s) were filtered using an FDR and adjusted p-value cut-off of <0.05. From this list, up and downregulated genes were distinguished using a logFC ≥1 and ≤-1 respectively. Significant DEG’s with a logFC <1 or >-1 were labelled as low expressed genes. All significant DEG’s were annotated using blastx and blastp against a manually curated UniprotKB database of Xcc proteins. For Trinity de novo supertranscripts, predicted longest ORF’s were validated using the blast2GO suite of OmicsBox.

### Gene ontology and pathway analysis

Insights into affected bacterial pathways were obtained using the Kyoto Encyclopedia of Genes and Genomes KEGG Pathway Mapper [27, 28]. Gene ontology (GO) and KEGG enrichment analysis were determined using hypergeometric distribution followed by FDR correction (0.05) via ShinyGo v0.61 [29] and annotations from String-db v10 [30]. Xcc BrA1 genome multi-fasta and annotation files reported by Ramnarine et al. (2022) [31] are provided via the zenodo repository [32] for cross-referencing, along with the Trinity assembled supertranscripts of filtered DEG’s.

### qPCR validation of differentially expressed genes from Xcc BrA1 RNA-seq data

Xcc BrA1 was cultured, and total RNA was extracted as outlined previously. qPCR primers were designed using PrimerBlast [33] from the gene targets (see Supplemental Table 2). The 5X All-In-One RT MasterMix (Bioland Scientific) was used to carry out cDNA synthesis from 500 ng of total RNA according to kit protocols. Each 20 uL qPCR reaction (Syber Green) reaction (2x qPCR Mix, Bioland Scientific) was carried out in triplicate (qTOWER 3 G). The following cycling conditions were used: 95°C for 5 minutes and 35 cycles (95°C for 30 s, 60°C for 30s). Initially, the *16s* rRNA, *gyrB* and *lrp* genes were assessed as suitable internal references using synthesized RNA-seq data and qPCR assessment. However, only the *lrp* gene was found to be suitable and was used as the normalization gene in further calculations. Ct values were obtained and analysed using a relative quantification approach via the Livak method [34]. Additionally, qPCR specificity was assessed using agarose gel electrophoresis.

### Xcc strain Cf4B1 culture conditions for qPCR co-expression study

Xcc Cf4B1 (NZ_JAFFQM000000000.1) was chosen from a collection of copper resistant local *Xanthomonas* isolates [31]. This strain represents another local isolate containing the Xcc BrA1 *cop* variant gene cluster [35] from a more recent isolation. To assess gene expression of *copLABMGF* and other target genes, Xcc Cf4B1 was revived over 48hrs on nutrient agar (NA) from glycerol stocks. Heavy metal salts were obtained from Sigma-Aldrich and used to make sterile 50mM stock solutions in DI water. Aliquots at the required concentration were made and added MGY broth before autoclaving. A 24hr active subculture was induced with 0.08 mM CuSO_4_.5H_2_O as outlined above. Two CuSO_4_.5H_2_O concentrations were chosen to assess expression after induction and are defined as the tolerant (0.8mM) and resistant (1.2mM) levels. Induced cultures were resuspended in 1.5 mL MGY broth amended at both levels of CuSO_4_.5H_2_O to a bacterial OD600 of 0.6. These were incubated as separate cultures per timepoint of 15 minutes, 1 and, 4 hr @ 35 ^0^C. Cells were then pelleted and immediately frozen in liquid nitrogen. Cells were treated in a similar manner to study the response of several efflux pump targets to toxic heavy metal exposure. Un-induced 24hr cultures were exposed to MGY amended with the following for 1 hr: 1mM ZnSO_4_, 1mM CuSO_4_.5H_2_O, 10mM Na_2_HAsO_4_.7H_2_O (As(v)), 1mM CdSO_4_, and 1mM CoCl_2_.6H_2_O. These toxic concentration limits were chosen as resistance stress levels based on a study by Romaniuk et al. (2018) [36]. Cells treated with unamended MGY broth were used as a calibrator sample in downstream analysis. Treatments were carried out in triplicate (biological) from separate culture vessels. Total RNA was extracted using TRIzol (Invitrogen) and samples were assessed as outlined above.

### Xcc Cf4B1 qPCR gene expression in response to heavy metal stress

The 5X All-In-One RT MasterMix (Bioland Scientific) was used to carry out cDNA synthesis and qPCR as outlined above. Cycling conditions consisted of 95 ^0^C for 5 minutes and 35 cycles (95 ^0^C for 30 s, 60 ^0^C for 30s). qPCR specificity was assessed using agarose gel electrophoresis in addition to melting curve analysis. Primers were designed using PrimerBlast [33] for the genes listed in Supplemental Table 3. The *lrp* gene was used as the internal reference. Obtained Ct values were analysed using a relative quantification approach via the Livak method [34]. Normalized gene expression values were transformed to Log2FC, with up and downregulation denoted using a Log2FC limit of 1. Low expression of genes was noted where Log2FC >-1 or <1. ANOVA was carried out on normalized data using IBM SPSS version 23 to evaluate significant differences in gene expression per gene within at each timepoint. Datasets for the copper and heavy metal exposure screens are given in Supplemental Tables 4 (raw Ct) and 5 (Log2FC) and, Supplemental Tables 6 (raw Ct) and 7 (Log2FC) respectively.

### Gene cluster organization and annotation

Genomic features of Xcc Cf4B1, including efflux pump genes, heavy metal resistance genes, mobile elements and plasmid derived contigs, were identified and analysed in a previous study [31]. Gene clusters containing target genes, primarily MDR efflux clusters, were further assessed using Uniprot [37], InterProScan [38], blastn and blastp [39] and MetalPDB [40]. This allowed proper classification of ambiguously annotated genes to the nearest Xcc RefSeq genome ATCC 33913 homologs, highlight conserved domains and determine if specific metal substrate binding sites were present in any predicted protein sequence. The organization of gene clusters were visualized using Gene Graphics [41].

## Results

### Transcript assembly metrics

Read counts, quality metrics and mapping rates are listed in Supplemental Table 1. The average mapping rate (HISAT2) against the reference genome for control and treated samples were 92% and 88% respectively. The Trinity De-novo approach gave 115,923 transcripts (average length = 317bp). However, a low mapping rate to transcripts (38-23%) resulted in 92,601 transcripts after filtering. Further filtering removed spurious transcripts that did not map to the BrA1 genome. Duplicate, significant DEG’s/ transcripts (EdgeR) from both reference-based, and de-novo approaches were removed if present and both datasets combined. A total of 2293 differentially expressed genes were identified and categorised based on logFC as upregulated (≥ 1), downregulated (≤ -1) or low expression (0.9 to -0.9) genes. Of these, 638 were upregulated (37 de-novo transcripts), 337 downregulated (21 de-novo transcripts) and, 1318 showed low expression (8 de-novo transcripts) (Supplemental Tables 8 - 10).

### Early transcriptomic response of Xcc BrA1 to copper stress

**Fig. 1:**
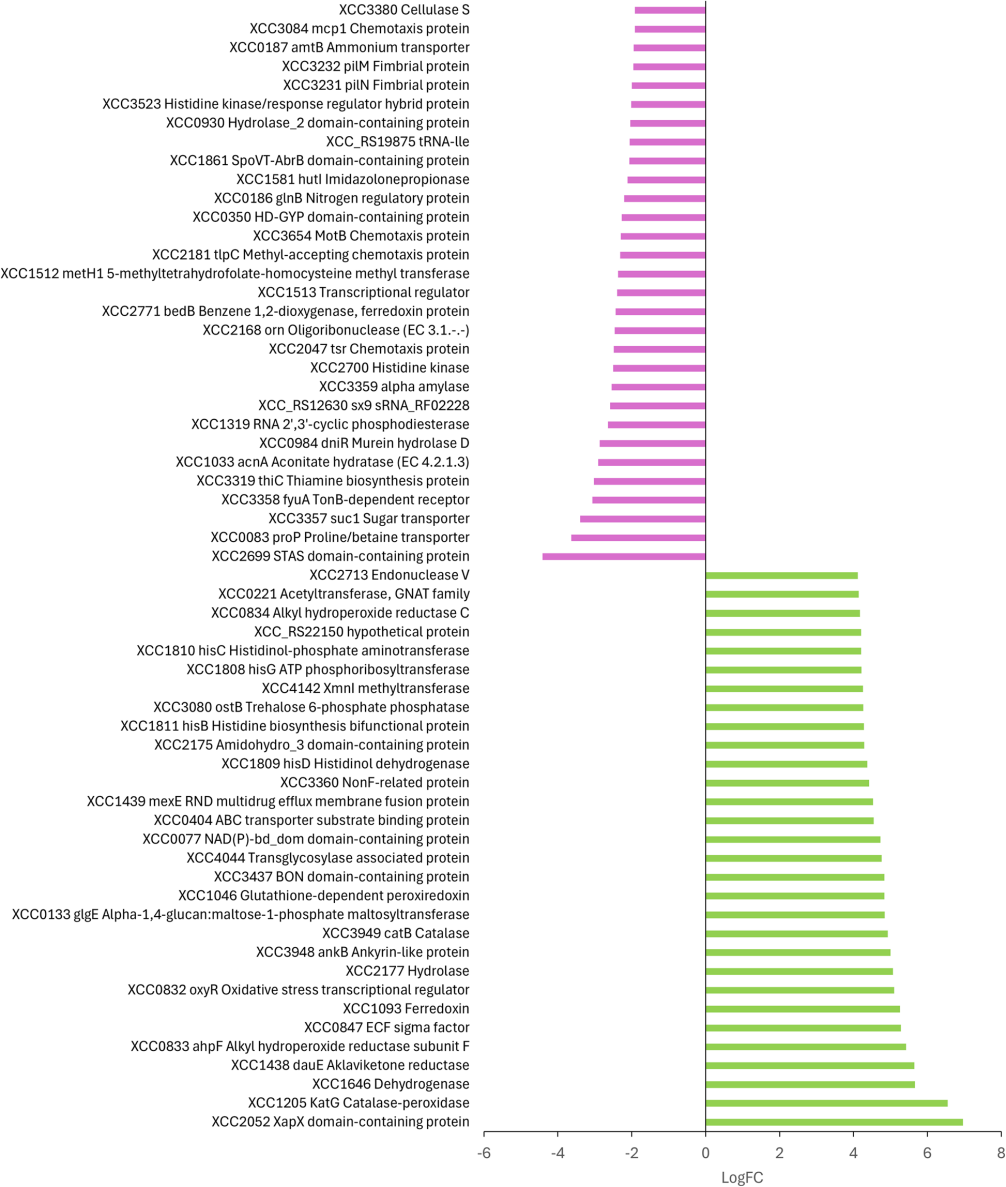
Top 30 significant DEG’s (logFC ≥1- ≤ -1) after 15 minutes of exposure to 0.8mM CuSo_4_.5H_2_O.

Figure 1. displays the top 20 up and downregulated genes, with a range in calculated logFC of 4.1 to 7 and -1.9 to -4.4, respectively. Upregulated genes were involved in histidine biosynthesis; phenylalanine, tryptophan and, tyrosine metabolism; oxidative stress response (*katG*, *ahpF*, *oxyR*, peroxiredoxin), iron transport and, transcriptional regulation (sigma factors). Other upregulated genes outside this range were the sX9 sRNA, cation transport (chromosomal *czcCBA*, *mntH*, *cutA*), *sod* superoxide dismutase, a *gsp*, EPS biosynthesis, *tonB* like protein, XCC_RS10740 orf112 phage related protein and IS1404 transposase and other mobile element proteins (Supplemental Tables 8 and 11). Downregulated genes in Fig. 1 were mainly involved with bacterial chemotaxis and regulatory genes in the two-component system, with other genes of interest involved in secretion, virulence pathways and *avr* response (Supplemental Table 9). Of note, uncharacterised proteins with no known function were differentially expressed (130 upregulated (logFC 1 to 5.7) and 40 downregulated (logFC -1 to -3.2). Additionally, other sX9 sRNA (Rfam: RF02228) genes, XCC2066 Phage-related protein, *cohL* (low +ve logFC) were upregulated while IS1477 transposase, *intS* phage-related integrase and *ihfAB* integrase host factor genes were downregulated. While counts were detected for *copLAB* genes and others in the same gene neighbourhood (contig), these genes were not significantly differentially expressed and did not pass the minimum CPM filter. Interestingly, genes directly flanking the copper homeostasis *cohLAB* operon were upregulated (PLP dependant cystathionine beta-synthase, oligopeptidase A and *gloI*) including elements of an upstream ABC-type exporter cluster, while the *fabAB* genes were downregulated and other genes in this locus showed low expression (Supplemental Table 10).

### The early response of Xcc metabolic pathways and systems to copper stress

**Fig. 2:**
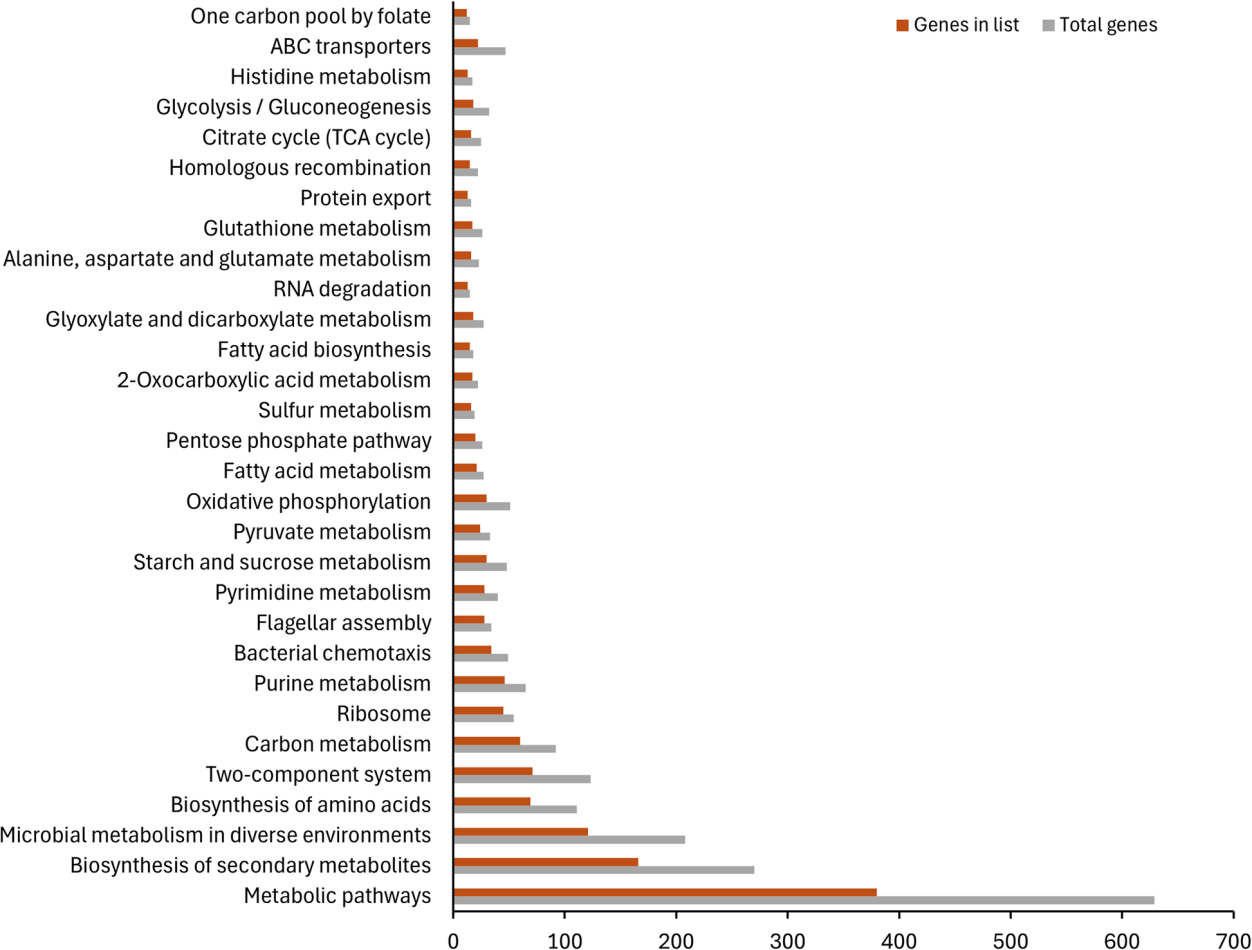
Enriched KEGG pathways of DEG’s. Enrichment of genes were calculated using the ShinyGo tool. The reference list refers to the total number of genes in KEGG pathway for the reference strain XCC ATCC 33913

Insights into affected pathways relating to all significant DEG’s and enriched gene ontologies were obtained from the KEGG Pathway Mapper and are presented in Supplemental Figure 1, Fig. 2 and Supplemental Tables 13-15. Enriched biological processes (BP’s) linked to upregulated genes fell into the categories of small-molecule metabolic and biosynthetic, oxidation-reduction, histidine metabolism and biosynthesis and, others. Enriched downregulated genes covered protein metabolism, nucleobase-containing compound metabolism, macromolecule and aromatic metabolism. Enrichment of molecular functions (MFs) mainly focused on cation/metal ion binding, oxidoreductase activity, cofactor binding, antioxidant binding and transferase activity. Downregulated genes were mainly linked to structural molecule activity, ribosome structure and binding, heterocyclic compound binding and, anion and ATP binding. These enriched BP’s and MF’s fell into the following KEGG pathways; Two-component systems, sulphur metabolism, protein export and bacterial secretion, oxidative phosphorylation, DNA repair and replication, fatty acid metabolism and biosynthesis, glutathione metabolism, amino acid biosynthesis and metabolism and bacterial chemotaxis, among others (see Fig. 2).

The KEGG BRITE hierarchical classification system further explored enriched KEGG pathways (Supplemental Table 12). Forty BRITE modules comprising all significant DEG’s were obtained but the following were focused on Enzymes (xcc01000), Transporters (xcc02000), Bacterial motility proteins (xcc02035), Secretion system (xcc02044), Transcription factors (xcc03000), Chaperones and folding catalysts (xcc03110), Two-component system (xcc02022), Lipid biosynthesis proteins (xcc01004), Lipopolysaccharide biosynthesis proteins (xcc01005), Prokaryotic defence system (xcc02048), DNA repair and recombination proteins (xcc03400), and DNA replication proteins (xcc03032) and, Transcription machinery (xcc03021). The broad enzyme module encompassed DEG’s classified as oxidoreductases acting on many groups and ions, peroxidases, transferases, hydrolases and translocases, ligases. The second broad grouping consisted of upregulated bacterial transporter genes functioning as ATP efflux pumps, transporters of: sulphate-thiosulphate, osmoprotectants, phospholipids, LPS and H+; ions channels for K+, glutathione, Mg, cobalt, zinc, phosphate, iron; as well as multi-resistance efflux pumps eg RND, *emrA* and *mex* genes.

The motility proteins *che*, *mcp*, *mot* and *tsr* were downregulated, except *cheB*, *cheY* and *tsr* XCC0324. Flagellar assembly proteins were mostly upregulated, except for *fliC*, while pilus assembly, fimbrial and twitching proteins (*pil* genes) were downregulated. General secretion system genes (*xps* and *xcs*) of the T2SS were upregulated especially *xcsM* and *N*. Low expression was observed with two *hrp* genes of the T3SS. One T4SS gene *virD4* was downregulated along with all *sec* system genes. Conversely, *tatA* and *C* of the TAT system showed low expression. Transcription regulators in the following families were upregulated; AraC, LysR and MarR. Others in the LacI, DtxR and PadR families showed the opposite trend. Genes in the GntR, TetR/AcrR, Lrp, LuxR, Fur, XRE, FrmR, and ParB families were expressed at low levels.

Of the chaperons and folding catalysts, genes encoding heat-shock proteins were upregulated including *clpA*, *hslU*, *hspA* and *hslR,* while chaperones such as *mucD* and *atpB* were downregulated. Protein disulphide thioredoxins (*trx* genes) and glutaredoxins (e.g., *grxD*) were upregulated. Two-component sensory (TCS) regulators related to redox response and an unclassified TCS gene were upregulated, but downregulation in 2 chemosensory genes (*pilG* and *H*) was seen. Low expression, however, was observed with TCS genes linked to TCA transport, carbon metabolism and regulation of lipid fluidity. Conversely, high expression was seen with phosphate starvation response and genes responsible for oxyanion binding.

**Fig. 3:**
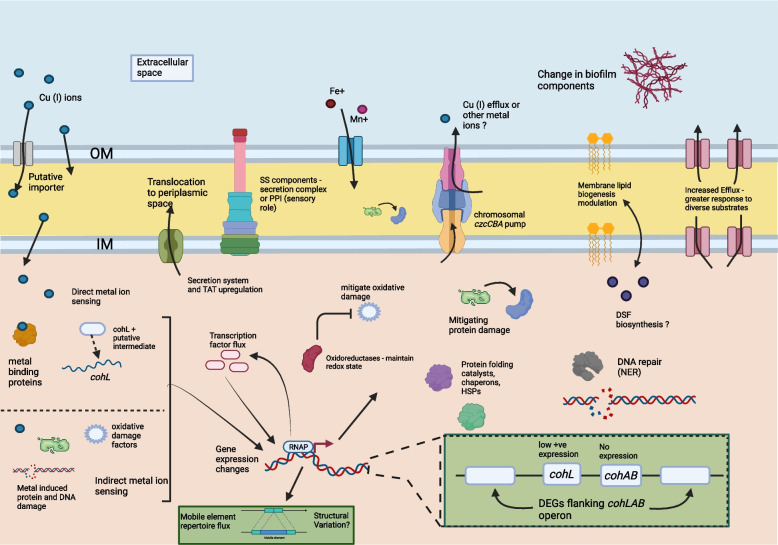
Modelled early responses to copper stress in Xcc BrA1 challenged by a 15-minute exposure to 0.8mM CuSO_4_.5H_2_O. OM – Outer membrane, IM – Inner membrane. Predicted functions are inferred as initial responses to copper stress. Image generated using Biorender.com

Most genes in the fatty acid biosynthesis pathway were downregulated (*fab* genes and acyltransferases). Two 3-oxyacyl-ACP reductase genes and roughly half of the LPS biosynthesis genes were upregulated. Genes involved in direct and base excision repair, AP nucleases in single-stranded breaks, the NHEJ DNA repair complex, translesion DNA synthesis, were upregulated. DNA polymerase I, polymerase III holoenzyme, ligase I, those in the *recFOR* pathway among others were downregulated. Furthermore, transcription terminators, elongation factors and core subunits of RNA polymerase were downregulated. Many sigma factors were upregulated (ECF sigma factor, RNA polymerase sigma factor 32 and 54). A summary of the early response of Xcc BrA1 to copper stress is illustrated in Fig. 3.

### qPCR validation of RNA-seq results using a subset of DEG’s

The randomly chosen genes for qPCR validation of the RNAseq data were: STAS, *katG*, *gyrB*, *xcsM*, AvrXccC1, *mcp1*, *hrpX*, EngXca, FusD, *oxyR*, *mdtA*, *cohB*, *cohA*, *cusA*, plasmid borne *czcB* and the chromosomal *czcB*. The qPCR Log2FC values obtained showed similar trends with RNAseq values with an R^2^ correlation of 0.589 obtained (Supplemental Figure 2). qPCR values for genes targets and related RNAseq Log2FC values are given in Supplemental Table 2.

### Target genes and their organization in the draft Xcc Cf4B1 genome

**Fig. 4:**
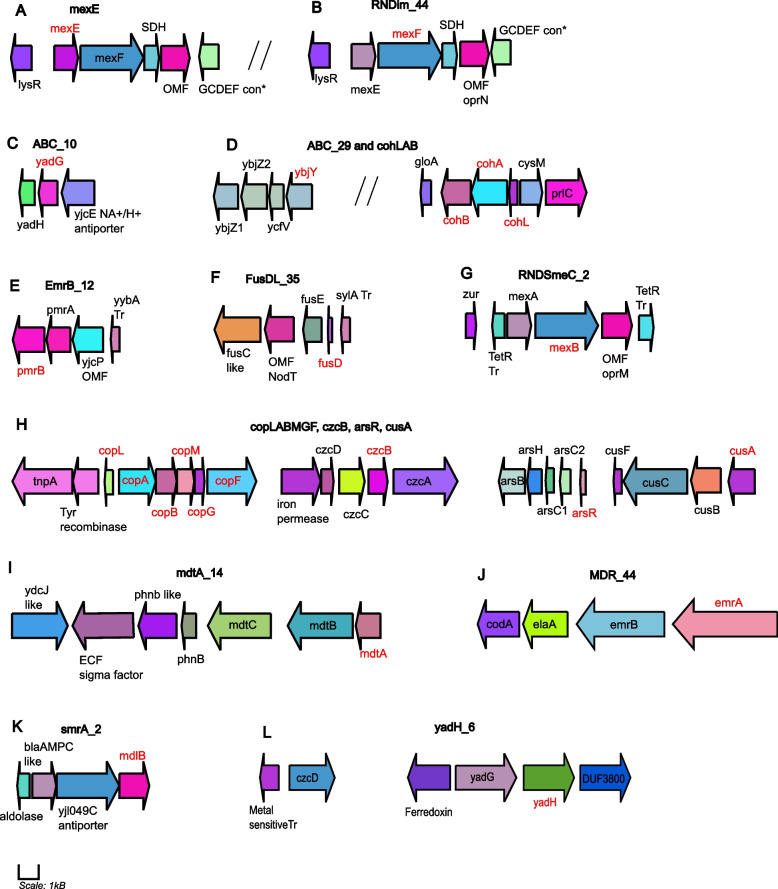
qPCR gene targets and respective gene cluster organization in Xcc Cf4B1. Primer target names are given at the top of each gene cluster diagram from A-L, and targeted genes for each respective primer are given in red text. Gene colours are default outputs and represent different CDS

Target genes outlined in Supplemental Table 3 and their corresponding gene cluster in Xcc Cf4B1 (NZ_JAFFQM000000000.1) are visualized in Fig. 4. Genes were annotated using RASTtk and Uniprot to assign the nearest best homolog protein and corresponding gene in the Xcc RefSeq genome (ATCC 33913). The *czc* cluster containing the *czcB* target is predicted to occur on a chromosomal contig (NZ_ JAFFQM010000094.1). This resolved contig containing this cluster was only 14.7 kb long with a single IS3 family transposase on one end and matched with >90% identity to complete Xcc chromosomes. MDR efflux clusters from the RND (4A, 4B, 4G, 4H [*czcB* and *cusA*], 4I [ABC_29]) and *czcB* (chromosomal), MFS (4E and 4J) and ABC (4C, 4D, 4F, 4K, 4L) superfamilies are represented in Fig. 4. Also depicted are the *copLABMGF* and *ars* operons (4H). Details on the MDR efflux gene clusters’ (4A-4L) annotated functions, efflux pump superfamily and predicted metal-binding sites are given in Supplemental Table 16. Complete (100%) coverage with 97-100% sequence ID across multiple NCBI Xcc genomes were noted for all gene clusters, except 1H, and with Uniprot entries matching genome annotations.

### Initial and late-stage expression of heavy metal resistance genes and putative metal responsive MDR efflux pumps in response to toxic levels of heavy metal

**Fig. 5:**
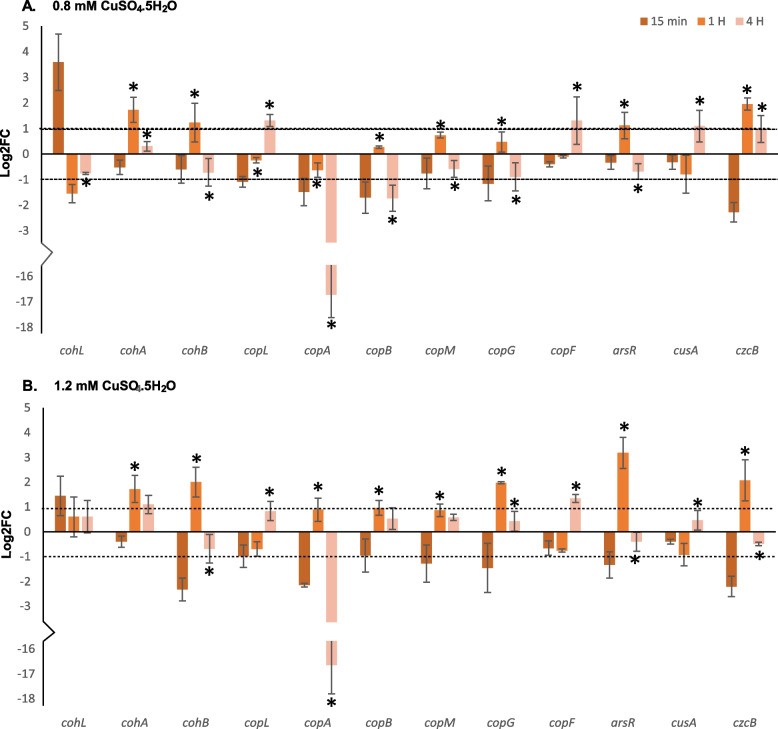
Upregulation of copper resistance (*cop*), homeostasis (*coh*) and other plasmid-borne heavy metal resistance genes in response to 0.8 mM (A) and 1.2 mM (B) CuSO_4_.5H_2_O stress levels. The dotted lines represent a Log2FC >=1 and <=-1 cut off used to denote a 2X change in gene expression. Asterixis (*) above bars for the 1 and 4 H timepoint represent a significant difference in Log2FC compared to the previous timepoint. Error bars represent standard deviation

**Fig. 6:**
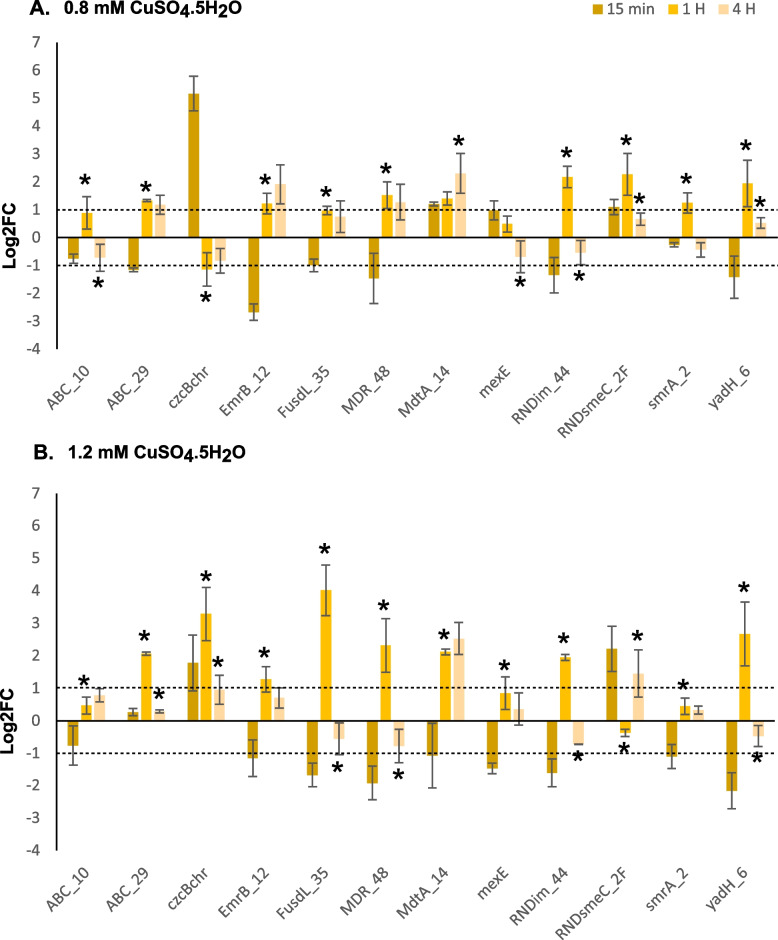
Upregulated expression profiles of efflux pumps in response to 0.8 mM (A) and 1.2 mM (B) CuSO_4_.5H_2_O stress levels. The dotted lines represent a Log2FC >=1 and <=-1 cut off used to denote a 2X change in gene expression. Asterixis (*) above bars for the 1 and 4 H timepoint represent a significant difference in Log2FC compared to the previous timepoint. Error bars represent standard deviation

Elevated expression at 0.8 mM and 1.2 mM CuSO_4_.5H_2_O concentrations at the initial (1 hr) and late-stage (4 hr) response was assessed in addition to a 15-minute timepoint meant to capture extreme expression responses. Gene expression at these timepoints for both concentrations and stress levels are given in are represented in Figs. 5 and 6, and values are given in Supplemental Table 4. Chromosomal homeostasis *cohA* and *cohB* were first upregulated at both stress levels at the initial stage with lowered expression/downregulation at the later stage. While this was more pronounced at 1.2 mM CuSO_4_.5H_2_O, *cohA* maintained upregulation as a late-stage response. Interestingly *cohL* was highly upregulated at 15 min but downregulated after at 0.8 mM. This trend was mostly seen at the higher stress level, but expression was maintained up to the late stage albeit < Log2FC of 1. Notably *cohA* and *B* were not expressed at the 15-minute time point in either treatment. Contrastingly, *copL* was upregulated only at the late stage for both concentrations and the *copA* and *B* genes were expressed at the initial stage at 1.2 mM only. Notably, *copA* was strongly downregulated at 4 hr. Further along in the operon, *copM* was upregulated at the initial stage for both stress levels but showed low expression only at 1.2 mM at the late stage. The *copG* gene showed similar expression patterns as *copM* at 0.8mM but showed greater upregulation at the initial stage at 1.2 mM. The *copF* gene showed similar expression patterns to *copL.* Interestingly the *cusA* homeostasis gene was upregulated only at the late stage at 0.8mM. Genes of the heavy metal resistance operons downstream of *copLABMGF* were also expressed under copper stress. The *czcB* gene was upregulated at both stress levels at the initial stage but expression extended into the late stage only at the 1.2 mM. The *arsR* gene was significantly upregulated in both treatments at the initial stage with greater expression at higher stress levels.

The chromosomal *czcB* gene and MDR efflux pumps were assessed for expression under both copper stress treatments (Fig. 6) concurrently with those gene targets represented in Fig. 5. At the 15 min timepoint at 0.8 mM CuSO_4_.5H_2_O, only *czcBchr, mdtA_14, mexE* and *RNDsmeC_2F* were upregulated. At 1.2 mM for this timepoint, only *czcBchr* and *RNDsmeC_2F* were upregulated. At 0.8 mM the *ABC_10, ABC_29, EmrB_12, FusdL_35, MDR_48, MdtA_14, RNDim_44, RNDsmeC_2F, smrA_2* and *yadH_6* genes were upregulated at the initial phase but only *ABC_29, EmrB_12, FusdL_35, MDR_48, MdtA_14* and *RNDsmeC_2F* showed expression into the late stage. Generally, for the 1.2 mM stress level the same expression patterns with greater upregulation of these genes was observed at both stages. There were some exceptions including FusdL_35 and *smrA_2* (lower expression) and, the *czcBchr* gene which was now upregulated at the 1.2 mM stress level. Notably the highest expression was observed at the 15 min timepoint with *czcBchr* at 0.8 mM. The efflux pumps with the highest upregulation were generally seen at the initial stage, with *RNDim_44, RNDsmeC_2F* and *yadH_6* at 0.8 mM and *czcBchr, FusdL_35* and *yadH_6* at 1.2 mM. Interestingly, the highest upregulation at the late stage for 0.8 mM was observed with genes *EmrB_12* and *MdtA_14* while this was seen with the *MdtA_14* and *RNDsmeC_2F* genes at 1.2 mM.

**Fig. 7:**
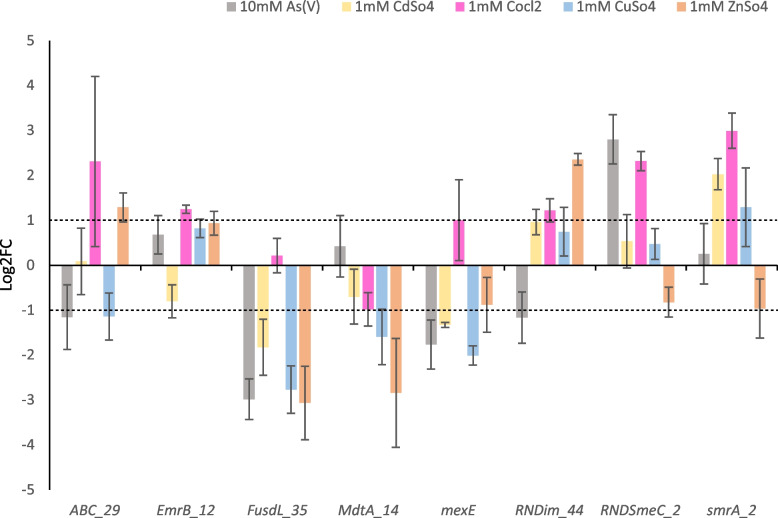
Effect of toxic heavy metal exposure on efflux pump gene expression at the initial stage. Error bars represent standard deviation

Efflux pump gene expression was further assessed under stress from different heavy metals at toxic concentrations (Fig. 7) to possibly ascertain whether the observed expression profiles seen previously were specific to CuSO_4_.5H_2_O. Of note, gene expression represented in Fig. 7 is from a separate subculture of the same strain that was not induced with 0.08 mM CuSO_4_.5H_2_O (Supplemental Table 5). All genes except *FusdL_35,* and *MdtA_*14 and *mexE*, were upregulated after exposure to different heavy metals at the initial stage. Notably, while *RNDim_44* was also upregulated after 1 mM CuSO_4_.5H_2_O exposure, the *smrA_2* gene showed the highest upregulation. This gene was also upregulated when exposed to CoCl_2_.6H_2_O and CdSO_4_. Other genes upregulated by CoCl_2_.6H_2_O were *ABC_29, EmrB_12, mexE, RNDim_44* and RNDSmeC_2. Those upregulated by CdSO_4_ were *RNDim_44* and *RNDsmeC_2*. The *EmrB_12, MdtA_14* and *RNDsmeC_2* genes were upregulated Na_2_HAsO_4_.7H_2_O and the *ABC_29, EmrB_12* and *RNDim_44* genes were upregulated by ZnSO_4_. The highest upregulation by each heavy metal is as follows; ZnSO_4_ = *RNDim_44*, CuSO_4_.5H_2_O = *smrA_2*, Na_2_HAsO_4_.7H_2_O = *RNDsmeC_2*, CdSO_4_ = *smrA_2*, and CoCl_2_.6H_2_O = *ABC_29*.

## Discussion

### Early transcriptomic response of Xcc BrA1 under copper stress

An exploratory study into early transcriptomic changes in Xcc to copper stress was carried out using a 15-minute exposure period. While broad responses to metal and oxidative stress are documented bacteria and studies often target specific genes, the early molecular cascade linked to copper stress response in *Xanthomonas* is not well characterised. A sub-toxic copper concentration for resistant *Xanthomonas* [42] was chosen to trigger homeostasis and tolerance responses. Overall, pathways involved in differential uptake and use of carbohydrates, chemosensory and motility, broad oxidative stress response through enzymatic activity, iron scavenging, biofilm formation, specific DNA repair mechanisms, mobile elements, protein folding and repair, metal efflux (not copper-specific), copper homeostasis (not *cohAB*) and expression of genes flanking the *cohLAB* operon were affected. A summary of these responses is modelled in the cell diagram in Fig. 3.

Early molecular responses do not appear to focus on copper homeostasis *cohAB* genes, however other metal binding and efflux genes (*czcCBA*, *mntH*, *cutA*) were upregulated at this early time point. Firstly, upregulation of the chromosomal *czcCBA* genes represents an intriguing response as plasmid homologs to this operon provide resistance to Co, Zn and Cd via efflux to the extracellular space [43]. The current study thus provides a putative association of this chromosomal cluster to a general heavy metal stress response which may be linked to oxidative stress, but further characterisation is needed. Other genes like *cutA* are involved in proper protein folding under metal stress in conjunction with *dsb* genes, both of which showed low expression [5]. Increased manganese uptake by *mntH* as opposed to efflux possibly by downregulation of *mnt* genes demonstrates a prepared state for metabolizing oxidative stress products in the cell [44]. Notably, no genes localized to the plasmid-borne putative composite Tn heavy metal resistance island were upregulated at this timepoint. Additionally, the broad oxidative stress response genes (*katG*, *ahpF*, *oxyR*) and numerous other oxidoreductases imply a greater metabolic shift towards mitigating oxidative damage first before efflux and other copper ion-specific mechanisms come into play.

Modification of the transcription factor (TF) repertoire shifted to those involved in heavy metal binding and responses to diverse environmental signals, setting the stage for a broad approach to the effects of toxic copper levels. Most were linked to general stress responses, pathogenesis, oxidative stress response and multi-drug resistance (MDR) etc in other bacteria [45]. The differentially expressed TCS (Two-component system) genes also reflect this fact, but no specific systems related to heavy metals were observed. Furthermore, ECF sigma factors like *rpoH* and *rpoN* are involved in heat shock protein expression and biofilm formation respectively [46, 47]. Generally, these factors are linked to broad responses including those relating to toxic environmental metal ions or iron depletion (*fecI*) [48]. Additionally, the downregulation of factors involved in translation elongation and affecting arrest of the RNA polymerase complex (*nus* and *gre* genes) further iterates the variable early effect on transcription [49, 50].

In conjunction with TFs, modulation of chemosensory genes, some linked to the flagellar motor system, involved in complex signal cascades responding to multiple ligands were also observed. This gene expression flux is most likely linked to secondary messenger profile changes and not necessarily only motility [51]. However, changes to flagellar motor function and biofilm due to differential expression of *cheY* homologs and other *che* genes are still inferred [52]. An apparent selection for TAT secretion over the Sec system was observed, but a specific result of this cannot be inferred as the TAT secreted proteins can show some degree of species-specific diversity in *Xanthomonas*. Additionally, upregulation of *xcs* T2SS genes further indicates the priming of cells’ ability to translocate proteins to the periplasmic space [53]*.* Interestingly, upregulation of a metalloprotein (oligopeptidase A) gene directly flanking the *cohLAB* operon is assumed to play a role in signal peptide degradation [54]. However, these proteins are highly conserved and associated with diverse physiological responses in bacteria [55]. Initial assembly of *vir* conjugation machinery is implied by low +ve expression while downregulation of the secretion-related gene *virD4* suggests a terminal movement of DNA/DNA-protein complexes to the periplasmic space which needs further investigation.

Upregulated heat shock proteins have the potential to diversify the intracellular response and have been documented in toxic metal stress responses in other organisms [56]. The differential expression of *clp* and *hsl* genes, involved in proteolysis maintenance, suggests a greater response towards protein unfolding and chaperon activity in response to heavy metal-induced damage and misfolding [57]. Pathways dealing with proper disulphide bond formation and elimination of erroneous occurrences (*grx* - glutaredoxins and *cut/dsb* genes) further supports this fact [5, 58]. Unregulated copper can also affect membrane components causing lipid profile changes under metal stress, possibly reflecting a shift towards more metal-protected forms [5]. Changes to outer membrane components via the Lipid A pathway in Xcc BrA1 support this. Furthermore, type II fatty acid synthesis was overall downregulated, including the *fabAB* cluster in the *cohLAB* locus. However, *fabG*, coding for a key enzyme that produces 3‐Hydroxyacyl‐ACP, was the only upregulated gene. This possibly reflects a shift towards DSF biosynthesis [59] in conjunction with membrane modification, which is further supported by the upregulation of *tesA*. This trend of changes to phospholipid metabolism linked to increased 3-Hydroxyacyl-ACP biosynthesis has been previously suggested as a metal stress response [60].

The initial flux of components between the cell and environment and the cytosol-periplasmic space is achieved through numerous transporters. The following processes were affected at the early timepoint: biofilm formation (*bapA*) [61], sulphate uptake and utilization (*cysU*, *nrtCD*) [62], xanthan biosynthesis, glutathione metabolism (*gst*) [63], enterobactin induced iron uptake (*befA*) [64], and metal, broad substrate efflux and MDR (*yadH*, *emrA*, *mexF*) [6, 65, 66]. These changes would prepare cells for oxidative stress, possibly xenobiotic resistance, redox homeostasis via cysteine/sulphated compound metabolism and possible efflux of copper or other metal ions via *czc* elements.

### Copper-induced gene expression of a plasmid-borne heavy metal resistance gene island in Xcc Cf4B1

In *Xanthomonas* spp., copper resistance is functionally encoded by the *cop* operon and the *coh* operon is assumed assist with copper tolerance and homeostasis [1]. In line with assumptions from that study, the *coh* operon was expressed prior to the plasmid borne *cop* operon. However, the former maintained expression up to 4 hrs in one case at the higher copper stress level. De Freitas et al. (2019) [3] showed that the *cohL* and *copL* genes are constitutively expressed at low levels. In the current study, the results suggests that the *coh* operon responds first to low levels of copper ions. When copper ion concentration increases to the resistant level, the *cop* operon is activated but the *coh* operon maintains expression. Co-expression up to 1 hr was observed but synergistic effects of both operons cannot be analysed from the approach used in this study. Furthermore, while protein levels were not measured, the downregulation of some genes in these operons (*coh/copA*) for instance does not automatically indicate a drop-in activity of their gene products. As copper binding and detoxification proteins, a maximal effect could have been achieved, early, after stress exposure and thus expression tapered off by 4 hrs [2, 67]. The expression patterns and predicted protein structures of *coh* and *cop* genes further suggests similar modes of action for gene products which are expressed at different times. Furthermore, predicted proteins for both *coh/copLAB* operons contain homologous domains indicating secretion from the cytosol or association with the outer-membrane. As such both are predicted to function in similar cellular niches. Thus, we propose that these two operons enable a stronger initial response sustained up to 4 hrs due to their apparent duplicity, but this must be confirmed.

A distinction in plasmid-borne *cop* operon activity and further added advantage of this gene cluster can be observed with the expression pattern and predicted function of the *copMGF* gene products. Increased resistance and survival under toxic copper stress are afforded by *copM* with uncharacterised effects caused by *copG* and *copF* [1]. The *copM* gene contains a cytochrome *c* domain, is required for full copper resistance [1] and may bind copper ions [68]. Recently, a *copG* homolog in *Pseudomonas* was characterised as a copper-binding oxidoreductase possibly involved in cycling *copA* dependant Cu (II) to Cu (I) for *cusCBA* efflux under anaerobic conditions [69]. As both *copM* and *copG* appear to be involved in binding copper reduced by *copA,* their late-stage downregulation might reflect a plateau in actively binding proteins which caused negative feedback on expression, similar to *copA.* The *copF* gene is predicted to be a P1B-Type ATPase and presumably moves copper ions from the cytosolic to the periplasmic space. As this gene was expressed only at the later stage in both copper stress levels, it can be assumed to serve as a secondary response to move toxic copper ions from the cytosol. Similarly, the expression pattern of *cusA* under both copper stress levels represents a later stage response. This gene functions under anaerobic conditions [70] which can compensate for the loss of *copLAB* function in oxygen-depleted states [71] which can occur under prolonged copper stress levels. This gene cluster provides an alternative route of Cu (I) ion removal from the cytoplasm and periplasmic space. These responses may occur due to diminishing or overwhelmed early molecular responses to copper stress and is noted to also occur at the lower stress level where other resistance genes were not expressed. The predicted model of *copLABMGF* activity in conjunction with other copper responsive elements such at the *cus* operon is given in greater detail in Supplemental Figure 3.

Within the heavy metal resistance gene cluster, only the *ars* operon contained its respective regulatory gene. It was assumed that the *czc* and *cus* operons were either under the transcriptional regulation of the *cop* operon’s *copL* or regulated by other means. Genes from these operons also showed upregulation at the resistant concentration. This implies that these heavy metal resistance genes may be part of a regulon within the local plasmid under the regulatory action of the *copL* gene. This gene expression pattern implies an increase in tolerance for multiple heavy metals (Co, Zn and Cd) upon exposure to copper, however, this may only occur when cells are induced at a very low and sub-toxic concentration. Functional validation of chromosomal *czc* operons in *Xanthomonas* is lacking. These genes may respond differently to resistance-associated *czc* homologs and are possibly part of a homeostasis mechanism [72]. Furthermore, in the case of the *ars* operon, upregulation by other heavy metals has been demonstrated in *Cupriavidus* [73]. Incidentally, a study on *Agrobacterium* arsenic resistance showed that *arsR* expression affected many global gene responses in that organism [74]. This may have implications in *Xanthomonas* as *ars* gene homologs are present in the local genome and might be part of the broad responses documented in the RNA-seq data. Induction via exposure to low concentrations of CuSO_4_.5H_2_O plays a key role in heavy metal resistance gene expression as they were not expressed in the second screen without induction. Induction might be a powerful driving factor in the survival of Xcc in fields where anecdotal reports from farmers indicate that the black-rot disease resurges after copper treatment. *Xanthomonas* spp. has the capacity to form dormant cells in the field under copper stress, where the reducing levels of copper agrochemicals after applications might allow for induction when senescent cells activate under alleviating stress conditions [75].

### Heavy metal induced MDR efflux pump expression in *Xanthomonas campestris *pv. *campestris*

Upregulation of 11 efflux pump gene clusters and a chromosomal *czc* operon were noted under toxic copper stress when strains were induced with a subtoxic level of CuSO_4_.5H_2_O. To determine if these genes responded to copper and other heavy metals without induction, expression was reassessed without this pre-treatment. Only 8 target genes were significantly expressed after exposure to various toxic heavy metal concentrations, but 3 showed low expression and the *czc* operon was not expressed. Efflux pumps consist of a large proportion of bacterial core genomes and are often functionally related, displaying high homology across bacterial genera. These pumps are thought to be ancestral features of bacterial genomes with recent evolution improving efficiencies towards substrates or narrowing specificities e.g., within the RND superfamily [14]. Their broad substrate activity and temporal expression suggest that bacteria employ these broad-acting systems in a specific manner as part of a global response to stress conditions, further highlighting their need for study in resistance phenotyping. While few clusters in the MDR family are responsible for heavy metal resistance, MDR efflux pumps usually allow for the removal of a broad variety of substrates from the cell cytoplasm [6, 76]. Interestingly, increased resistance to antibiotics in bacterial pathogens under heavy metal stress linked to metal-responsive efflux pump gene expression has been documented [11, 12].

Of the 11 MDR pump clusters targeted in this study, 4 are of the RND superfamily with one additional *czcCBA* chromosomal cluster was also within this family. Tripartite RND efflux proteins are prominent systems in Gram-negative bacteria and are involved in the efflux of many substrates including heavy metals (HME-RNDs) [4]. These proteins consist of the RND protein localized to the cellular membrane, and the MFP and OMF which forms the channel connecting the system to the outer membrane environment and thus provides outward movement from both the cytoplasm and periplasm. Two well-studied systems are the *czcCBA* efflux cluster involved in resistance to Co, Cd and Zn and the *cusCBA* cluster involved in resistance to Cu and Ag [4, 77]. The RND clusters in this study were homologs of *mexEF, mexAB* and *mdtABC* gene clusters which have not been directly associated with heavy metal efflux, however *mexAB* does contain *czcAB* like domains [6]. Furthermore, the *yadGH, macAB* and *mdlB* ABC and *emrAB* MFS pumps are also not associated with heavy metal efflux.

Despite this, all efflux pumps, except those targeted by ABC_10 and *mexE* primers, and the *czcB* gene, were upregulated at 0.8 mM CuSO_4_.5H_2_O and, all except the *mexE* target were upregulated at higher concentrations when cells were induced. The strong response of the chromosomal *czcB* gene to high CuSO_4_.5H_2_O stress levels up to the late stage is notable and this gene cluster represents an interesting target for future copper resistance phenotyping. MDR efflux gene expression is severely contrasted with the second evaluation using multiple heavy metals without pre-induction. The predicted metal bindings sites suggests that these targets are upregulated to increase stress response, and antibiotic and biocide resistance which has been noted in other bacteria on exposure to Zn concentrations [11].

On exposure to toxic metals without preinduction, only 5 of the 11 efflux pumps were upregulated with 3 of these affected by multiple heavy metals. The absence of preinduction allowed the elucidation of more direct responses as opposed to co-selection mechanisms, although this possibility cannot be ruled out. Greater weight was placed on pumps that responded to metals aside from Zn, as this ion was seen in other studies to upregulate other MDR pumps that were not related to metal efflux. Based on this criterion, only the ABC_29, RNDsmeC_2 and smrA_2 targets appear to be more directly associated with metal stress response. Notably only the smrA_2 target responded significantly to copper stress. The ABC_29 gene cluster was identified as a *macAB* homolog linked to macrolide antibiotic efflux [78] and the RNDsmeC_2 cluster was identified as a *mexAB-tolC* cluster with homology to metal binding *czcAB* genes. This cluster was initially identified as a low homology hit to the *smeDEF* gene cluster from *S. maltophilia*. A *smrA* homolog from an environmental *X.* spp. was shown to contribute to copper tolerance and was initially used to identify the SmrA_2 target [7]. This gene has been characterised as an *mdlB* homolog that contains a downstream cation/proton antiporter and a *blaAmpC* gene homolog. The function of *mdlB* on its own it not well understood [10] although the *mdlAB* cluster and homologs are involved in drug efflux jointly [79]. In the local strain genomic context, it appears that *mdlB* may be involved with drug efflux activity especially due to its association with a *blaAMPC* homolog. It has been shown that heavy metals can induce broad global stress responses in bacteria [12, 74]. However, the linking of an *sme* homolog in *Xanthomonas* spp. to both copper and antibiotic resistance [7] gives support for considering these MDR targets as part of the global response to heavy metals in *Xanthomonas*. Thus, these 3 efflux gene clusters represent important targets for further metal resistance investigation based on their response to toxic Cu, Co, Cd and As(V) exposure.

## Conclusion

Broad initial responses to copper stress were numerous and focused on changes in membrane lipid components, general stress responses and redox state homeostasis, protein folding catalysts and maintenance of disulphide bond formation, biofilm biogenesis, efflux pathways with diverse targets and DNA repair among others. Overall, the early responses to copper stress appear to focus on mitigating protein and downstream oxidative damage while priming cells for increased resistance to other metals and xenobiotics. Notably, only the *cohL* gene but not the *cop* operon was expressed at 15 minutes.

The sustained expression of *cohLAB* with later expression of *copLAB* genes under higher copper stress levels were seen. Furthermore, the later expression of the *copMGF, cusA* and possibly a chromosomal *czcB* genes provide putative evidence of copper ion efflux out of the cytoplasm as both a tolerance and resistance response. Further adding to the layers of resistance co-selection, many RND, ABC and MFS pumps linked to antibiotic efflux were also upregulated at increasing copper stress levels.

This study provides putative evidence of initial molecular responses to copper stress Xcc strain thus providing a baseline for further targeted studies that aim to explore specific homeostasis mechanisms in this species. The findings also highlight the broad resistance efflux mechanisms upregulated in a short space of time under copper/oxidative stress. The implication of which, seem profound in light of multi-resistant organisms and their impact on disease management and even human health. The findings outlined in this study was used to form a more targeted approach investigating the response of the heavy metal resistance island, chromosomal tolerance elements, MDR efflux pumps and to search for homologs of an acquired efflux systems. From this study, at least one putative copper responsive chromosomal gene cluster and several heavy metal efflux genes previously unlinked to these phenotypes were identified.

### Supplementary Information

**Additional file 1:** **Supplemental Figure 1.** Enriched KEGG biological processes (A) and molecular functions (B) of significant DEGs. The reference list refers to the total number of genes in each metabolic grouping from the Xcc ATCC 33913 reference strain. **Supplemental Figure 2.** Correlation plot of RNAseq and qPCR Log2FC values of 17 genes selected for validation.**Supplemental Figure 3.** A predicted model of the functional organisation of copper tolerance and resistance genes in local *Xanthomonas* isolates with elevated copper ion levels.The image was created using BioRender.com. The exact difference between *coh *and *cop *gene function is not known but may be regulated by different intracellular Cu ion thresholds. This figure summarises the complex interplay of copper plasmid-borne copper resistance and tolerance elements and the Cut family of proteins. *copA_H* refers to the *cueR* paired *copA* P-type ATPase, the CopF protein may serve a similar role. All *copLABMGF* localisations and functions are based on literature experimental evidence and, protein structural and domain characteristics determined from In-silico analysis of *cop *and *coh *genes in local Xanthomonas isolates characterised in Ramnarine, Jayaraj and Ramsubhag (2022).

**Additional file 2:** **Supplemental Table 1.** Read quality and mapping statictics of Illumina reads from copper stress and control treatments involving Xcc BrA1. **Supplemental Table 2.** qPCR primers and target genes for validation of Transcriptomic results. **Supplemental Table 3.** qPCR primer gene targets. Targets were identified in Xcc BrA1 from genomes characterised in Ramnarine, Jayaraman and Ramsubhag (2022). Ramnarine, Stephen D. B., Jr., Jayaraj Jayaraman, and Adesh Ramsubhag. 2022. "Comparative genomics of the black rot pathogen Xanthomonas campestris pv. campestris and non-pathogenic co-inhabitant Xanthomonas melonis from Trinidad reveal unique pathogenicity determinants and secretion system profiles." PeerJ 9 (e12632). **Supplemental Table 4.** Ct values for qPCR experiments on CuSo4.5H2O exposed cells at 15 (C15), 60 (C60) and 240 (C240) mins. Concentrations are given as 0p - 0ppm/0mM, 200p - 200ppm/0.8mM and 300p - 300ppm/ 1.2mM. **Supplemental Table 5.** Average log2FC values of gene targets under tolerant and resistant copper stress at 15 min, 1 hr and 4hr exposure time points. **Supplemental Table 6.** Ct values from qPCR experiments where by cells were exposed to different heavy metal concentrations for 1 hr. **Supplemental Table 7.** Average Log2FC values for MDR efflux pumps and other targets under heavy metal stress at the 1hr exposure. **Supplemental Table 8.** Upregulated genes (logFC ≥ 1). **Supplemental Table 9.** Downregulated genes (logFC ≤ 1). Supplemental Table 10. Low expression genes (logFC < 1 or > -1). **Supplemental Table 11.** Differentially expressed mobile elements, IS and Tn elements at the initial timepoint sampled. Refer to linked multifasta and annotation files to correlate with GeneID. **Supplemental Table 12.** KEGG BRITE categories summarizing significantly enriched DEGs (up, downregulated and low expression). **Supplemental Table 13.** Enriched KEGG Pathways (ShinyGO). **Supplemental Table 14. **Enriched Biological Processes (BP) GO terms (ShinyGO). **Supplemental Table 15.** Enriched Molecular Function (MF) GO terms (ShinyGO). **Supplemental Table 16.** Gene cluster classification, predicted function and probable metal-binding sites.

## Data Availability

All data is discussed in the manuscript, linked or presented in the Supplemental Tables and Figures files.
