# Supplementary material for: Early transcriptional changes of heavy metal resistance and multiple efflux genes in Xanthomonas campestris pv. campestris under copper and heavy metal ion stress
Source: BMC Microbiol. 2024 Mar 9;24:81. doi: 10.1186/s12866-024-03206-7 (PMC10924375; doi:10.1186/s12866-024-03206-7)
Supplement: Supplementary file 1 — Additional file 1: Supplemental Figure 1. Enriched KEGG biological processes (A) and molecular functions (B) of significant DEGs. The reference list refers to the total number of genes in each metabolic grouping from the Xcc ATCC 33913 reference strain. Supplemental Figure 2. Correlation plot of RNAseq and qPCR Log2FC values of 17 genes selected for validation.Supplemental Figure 3. A predicted model of the functional organisation of copper tolerance and resistance genes in local Xanthomonas isolates with elevated copper ion levels.The image was created using BioRender.com. The exact difference between coh and cop gene function is not known but may be regulated by different intracellular Cu ion thresholds. This figure summarises the complex interplay of copper plasmid-borne copper resistance and tolerance elements and the Cut family of proteins. copA_H refers to the cueR paired copA P-type ATPase, the CopF protein may serve a similar role. All copLABMGF localisations and functions are based on literature experimental evidence and, protein structural and domain characteristics determined from In-silico analysis of cop and coh genes in local Xanthomonas isolates characterised in Ramnarine, Jayaraj and Ramsubhag (2022). [file 12866_2024_3206_MOESM1_ESM.docx]

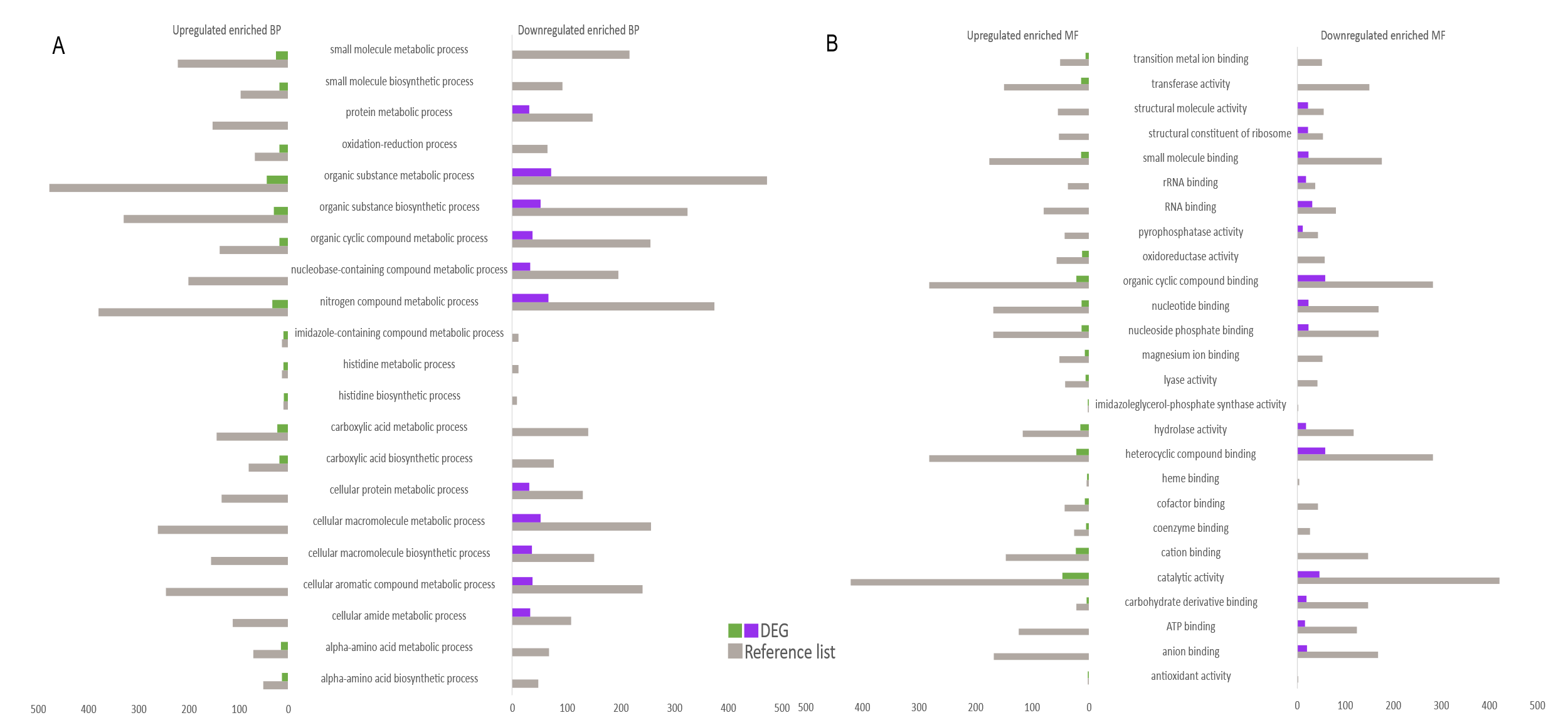


Supplemental Figure 1. Enriched KEGG biological processes (A) and molecular functions (B) of significant DEGs. The reference list refers to the total number of genes in each metabolic grouping from the Xcc ATCC 33913 reference strain.


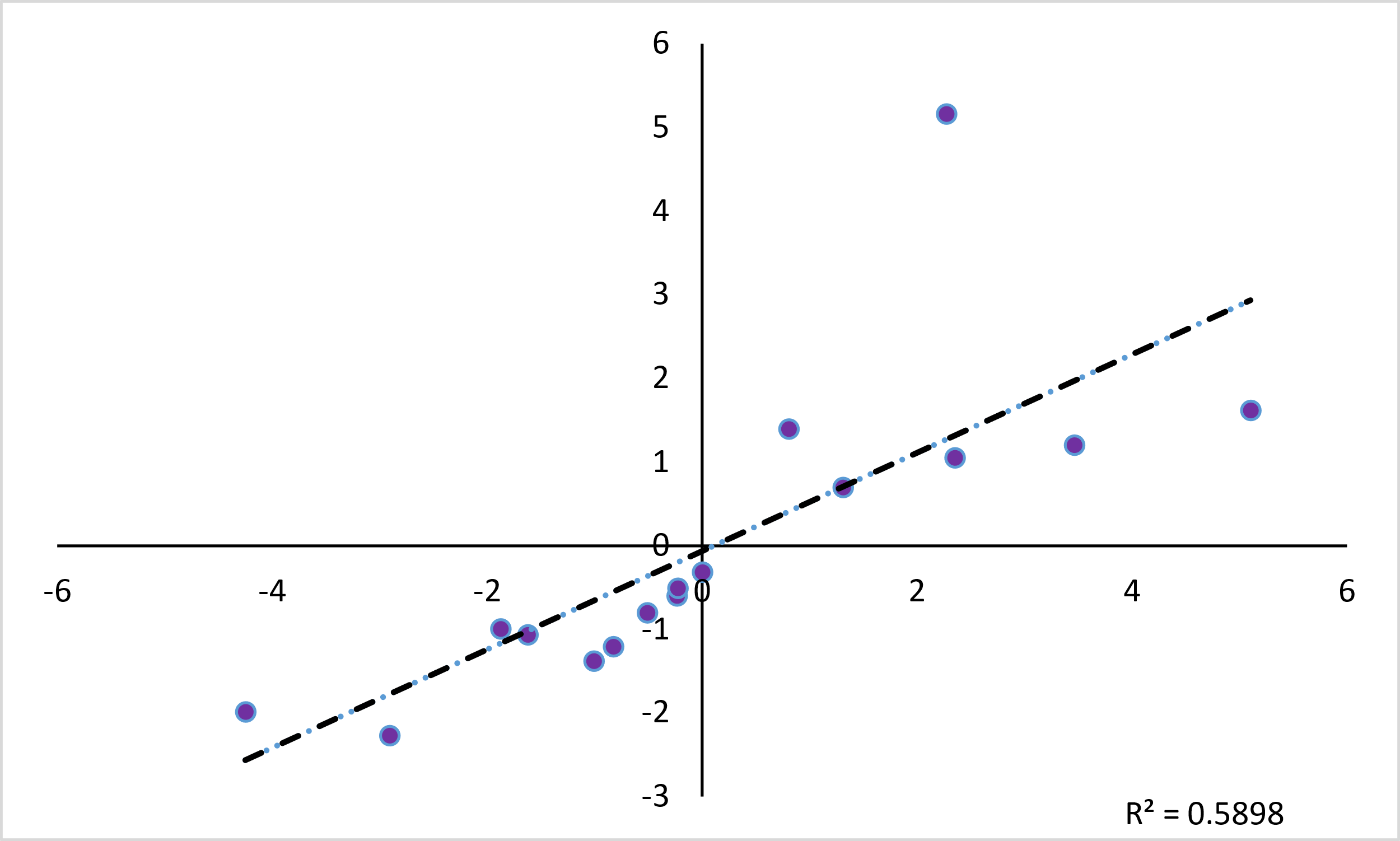


qPCR Log2FC

RNAseq Log2FC

Supplemental Figure 2. Correlation plot of RNAseq and qPCR Log2FC values of 17 genes selected for validation.


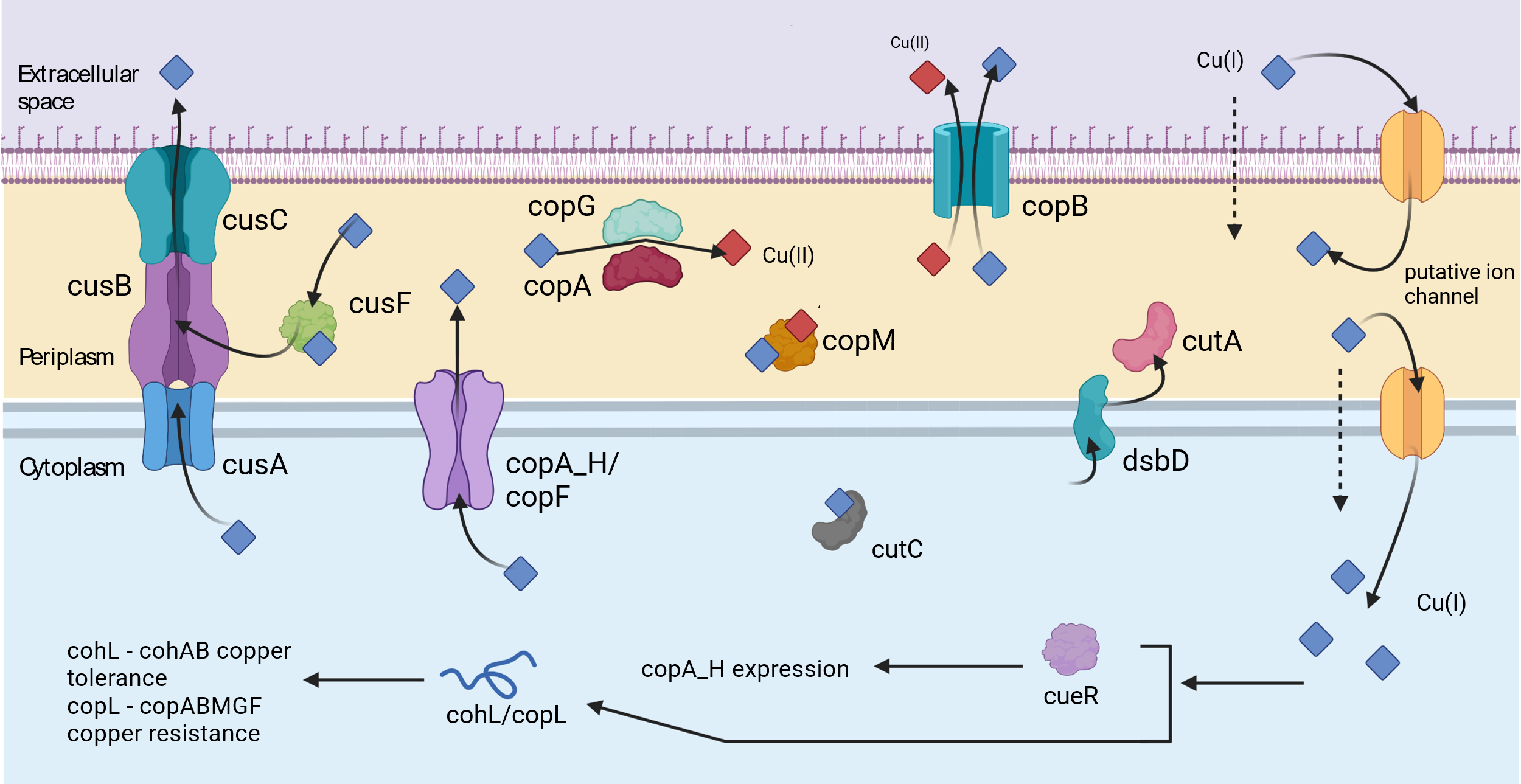


Supplemental Figure 3. A predicted model of the functional organisation of copper tolerance and resistance genes in local *Xanthomonas* isolates with elevated copper ion levels. The image was created using BioRender.com. The exact difference between *coh* and *cop* gene function is not known but may be regulated by different intracellular Cu ion thresholds. This figure summarises the complex interplay of copper plasmid-borne copper resistance and tolerance elements and the Cut family of proteins. *copA_H* refers to the *cueR* paired *copA* P-type ATPase, the CopF protein may serve a similar role. All *copLABMGF* localisations and functions are based on literature experimental evidence and, protein structural and domain characteristics determined from In-silico analysis of *cop* and *coh* genes in local Xanthomonas isolates characterised in Ramnarine, Jayaraj and Ramsubhag (2022).
